# Solving the influence maximization problem reveals regulatory organization of the yeast cell cycle

**DOI:** 10.1371/journal.pcbi.1005591

**Published:** 2017-06-19

**Authors:** David L. Gibbs, Ilya Shmulevich

**Affiliations:** Institute for Systems Biology, Seattle, Washington, United States of America; Oxford, UNITED KINGDOM

## Abstract

The Influence Maximization Problem (IMP) aims to discover the set of nodes with the greatest influence on network dynamics. The problem has previously been applied in epidemiology and social network analysis. Here, we demonstrate the application to cell cycle regulatory network analysis for *Saccharomyces cerevisiae*. Fundamentally, gene regulation is linked to the flow of information. Therefore, our implementation of the IMP was framed as an information theoretic problem using network diffusion. Utilizing more than 26,000 regulatory edges from YeastMine, gene expression dynamics were encoded as edge weights using time lagged transfer entropy, a method for quantifying information transfer between variables. By picking a set of source nodes, a diffusion process covers a portion of the network. The size of the network cover relates to the influence of the source nodes. The set of nodes that maximizes influence is the solution to the IMP. By solving the IMP over different numbers of source nodes, an influence ranking on genes was produced. The influence ranking was compared to other metrics of network centrality. Although the top genes from each centrality ranking contained well-known cell cycle regulators, there was little agreement and no clear winner. However, it was found that influential genes tend to directly regulate or sit upstream of genes ranked by other centrality measures. The influential nodes act as critical sources of information flow, potentially having a large impact on the state of the network. Biological events that affect influential nodes and thereby affect information flow could have a strong effect on network dynamics, potentially leading to disease. Code and data can be found at: https://github.com/gibbsdavidl/miergolf.

## Author summary

The Influence Maximization Problem (IMP) has been applied in fields such as epidemiology and social network analysis. Here, we apply the method to biological networks, aiming to discover the set of regulatory genes with the greatest influence on network dynamics. Fundamentally, since gene regulation is linked to the flow of information, we framed the IMP as an information theoretic problem. Dynamics were encoded as edge weights using time lagged transfer entropy, a quantity that attempts to quantify information transfer across variables. The influential nodes act as critical sources of information flow, potentially affecting the global network state. Biological events that impact the influential nodes and thereby affecting normal information flow could have a strong effect on the network, potentially leading to disease.

## Introduction

In order to respond to messages and environmental changes, cells dynamically process information arriving from cell surface receptors [1,2]. Information is transferred, stored, and processed in the cell via molecular mechanisms, often triggering a response in the regulatory program. These types of dynamic genetic regulatory processes can be modeled and analyzed using networks.

**Fig 1:**
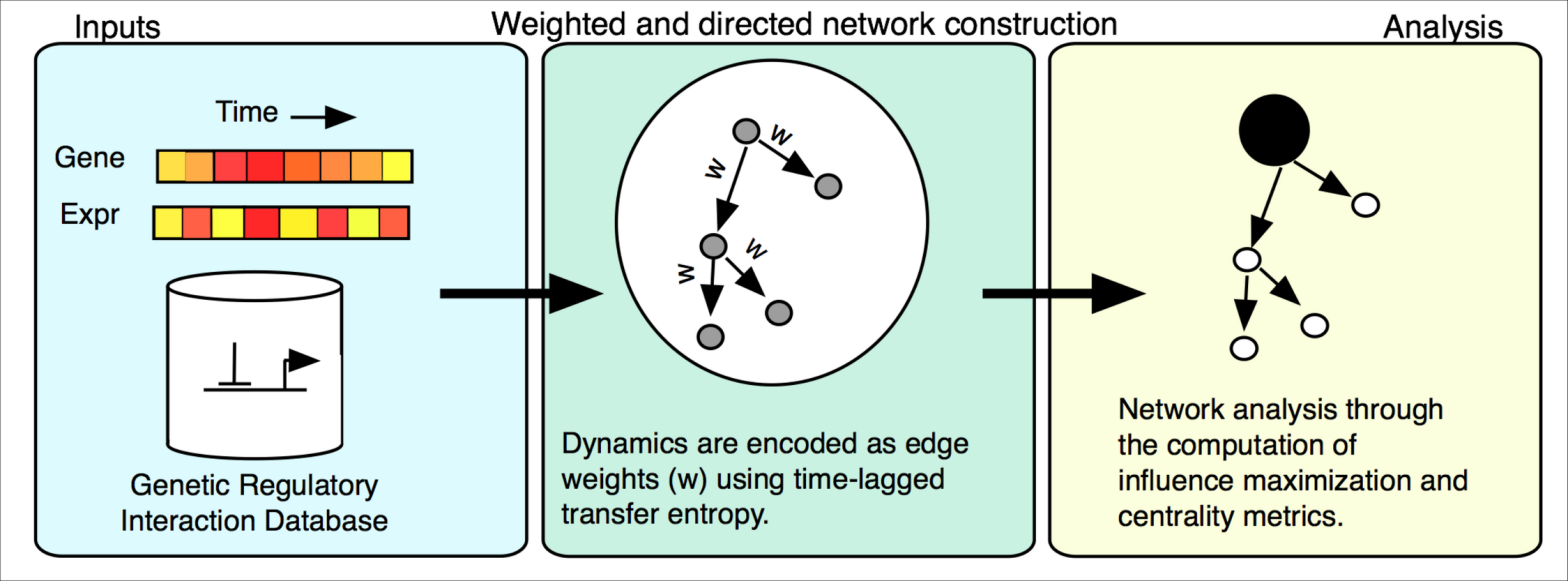
Analysis workflow. All regulatory edges from the YeastMine DB formed the regulatory network scaffold. Using time series gene expression data, time lagged transfer entropy was calculated and each edge was evaluated using a permutation-testing framework. The resulting network was used for solving the Influence Maximization Problem.

The cell cycle process in *Saccharomyces cerevisiae* is well studied, but not completely characterized [3]. The dynamic regulatory process is controlled by a network that processes signals. To gain further understanding of the regulatory structure, we used publicly available time series data and regulatory databases to solve the influence maximization problem (IMP) (Fig 1) [4,5].

Recently, the influence maximization problem (IMP) has received a great deal of interest in social network analysis and epidemiology as a general method for determining the relative importance of nodes in a dynamic process [6,7]. Use case examples are found in modeling the spread of infectious disease in social networks and in identifying optimal targets for vaccination (or advertisements) [8]. The IMP is a search over sets of nodes that, when acting like sources in a diffusion process, cover as much of the network as possible [9,10].

Diffusion on graphs is part of a general class of problems where some quantity flows from source nodes, across the edges of a graph, draining in sink nodes. Various forms of network flow methodologies have found success in algorithms such as Hotnet, ResponseNet, resistor networks, and others [11,12,13]. Diffusion, like the propagation of infection, does not follow algorithmically defined paths on graphs, such as shortest paths, but instead flows on all possible paths. In this work, we use a diffusion algorithm that is modeled using a random walk, where transition probabilities are proportional to edge weights. The random walk produces an expected number of visits to each node. If the expected number of visits is greater than a given threshold (here 0.0001), the node is considered to be ‘covered’, and the network cover is a count of ‘covered’ nodes. The goal of the IMP is to maximize this network cover with a fixed number of nodes.

In our application of the IMP to genetic regulatory networks, the diffusion process represents a flow of information on the network, which opens up many applications in biology [14,15,16]. Directional information flow can be described quantitatively using the model free method, transfer entropy (TE) [15]. Since processes in biology are not instantaneous, time lags are introduced, representing a lag between the transmission and reception of information. As an example, the expression of transcription factors, their subsequent binding to promoter regions, and ultimately, the induction of transcription can take substantial amounts of time.

In this case, we use ant optimization to search for sets of source nodes that lead to diffusion generated network covers that score highly [17]. Typically, ant optimization is used for path finding, but it can also be applied to combinatorial, subset selection problems [18,19]. In ant optimization, ants construct potential solutions as sets, which are scored and reinforced, encouraging good solutions in later iterations. In this work, the result of the optimization procedure is an optimal, or nearly optimal, set of nodes that maximizes network cover after applying the diffusion [15]. In application to biological networks, the IMP essentially remains an unexplored area of research [20].

Each run of the IMP returns a solution set of size *K*. Using both ‘fast’ and ‘slow’ parameter sets for the ant optimization, we have run the IMP for values of *K* from 1 to 50, resulting in 50 solutions, one set for each value of *K*. Genes were ranked by counting the number of times a given gene appeared in a solution set. A highly influential gene would appear in the solution for many values of *K*, regardless of the solution set size, implying that topologically, the gene is in an optimal position as a source of information, enabling contact to a large portion of the network. Optimization can proceed at different rates; more restarts, more ants, a slow pheromone evaporation rate, and a high number of local optimization steps may result in more robust and repeatable results, but more iterations might be needed and the run time can be longer. On the other hand, few restarts with a small number of ants and a fast evaporation rate, plus fewer local optimization steps, leads to more stochastic results and a shorter run time. The slow-and-steady approach can consistently get stuck in non-optimal minima, whereas the highly stochastic results can sometimes 'jump' out of non-optimal minima. In order to explore results and convergence behavior, both fast and slow parameter sets were used. Our results from either parameter set were in excellent agreement regarding influence rankings, reducing concerns about the stochastic nature of ant optimization.

To better understand topologically where the influential genes are situated, we compare the IMP solution sets to gene sets derived from other centrality metrics, such as degree centrality [21], betweenness-centrality [22], where shortest paths are considered, and PageRank [23], the algorithm used in web search.

This analysis produced a ranked list of genes that agrees with previous studies of cell cycle regulators and models, giving credence to the method as a fairly general approach to analyzing large scale biological network dynamics.

## Results

### Statistical network construction using time lagged transfer entropy

The yeast genetic regulatory network was constructed starting with 26,827 genetic regulatory edges from YeastMine and statistically filtering out edges [4]. Regulatory processes in biology are not instantaneous, so time lags are introduced to account for propagation time (S1 Fig) [24]. Further, genetic regulatory interactions are directional; transcription factors act on genes, and not the other way around. So, although correlation is easy to compute and is sometimes used to estimate the activity of regulatory edges, there are more appropriate metrics to use with time series data, such as transfer entropy. Transfer entropy (TE) is a model-free method that attempts to quantify information transfer between two variables in a directional manner. At present, computing transfer entropy is not trivial, and there is active research in comparing and deriving methods for approximating the value. In this work, we used a Gaussian kernel density based approach, which has been previously shown to be relatively accurate [25,26].

Using time series data for 5,080 measured genes and 26,827 genetic regulatory edges from YeastMine, both time lagged Spearman's correlation and transfer entropy were computed for all regulatory edges. Permutation-based statistics were applied to assess the significance of TE. Edges were accepted if empirical p-values was less than or equal to $\frac{1}{\left(p_{n}+1\right)}$ where *p*_*n*_ is the number of permutations (*p*_*n*_ = *50*,*000*).

Spearman's correlation tests were performed on each time lag (0–5 time steps). The maximum *ρ* was kept, and at FDR 1%, this resulted in 12,555 edges, containing 3,939 nodes. Significant edge weights had a median correlation of 0.58. Most of the edges (52%) showed a maximum correlation when using a time lag of zero.

**Fig 2:**
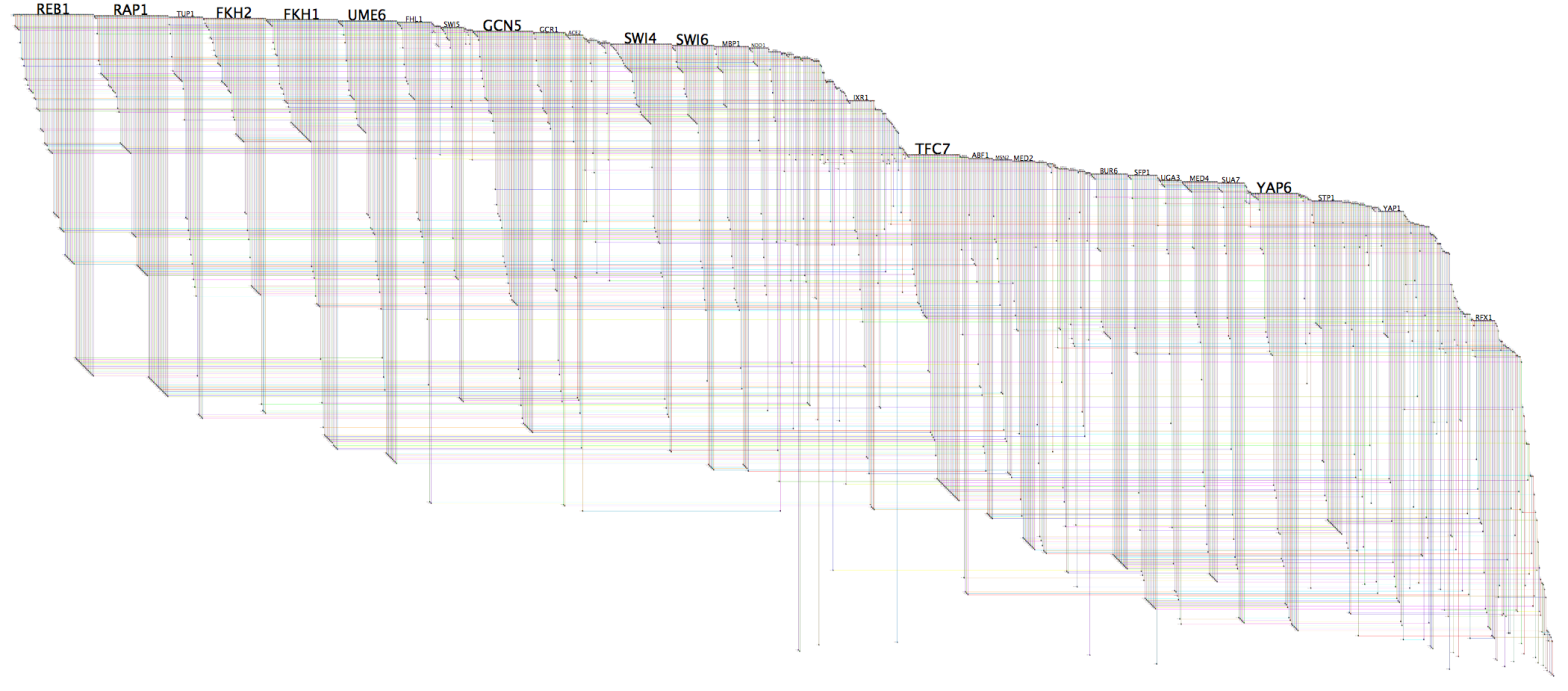
The resulting cell cycle network after significance testing. BioFabric representations are a novel way to visualize graphs. The depiction shows each node as a unique horizontal line and each edge as a unique vertical line. This makes some network structures easy to visualize. For example, high degree nodes can be seen as ‘wedges’ in the graph (nodes with out-degree 0, and in-degree 1, have been filtered out).

The metric of interest, time lagged TE, resulted in 2,084 significant edges containing 1,409 nodes with median values of 0.499 (Fig 2).

The overlap between the correlation and TE networks is moderate; only 16% of the edges in the correlation network are shared with the TE network (1,988 of 2,084 edges in the TE network or 97%), and while most TE nodes are found in the correlation network (95%), only 35% of the correlation nodes are found in the TE network. When comparing Spearman's and TE weights on matched edges, the correlation between matched edge weights was moderately weak (Spearman's correlation 0.43). Additionally, the mean node degree distribution in the correlation network is much higher than that of the TE network. For example, SFP1 has degree 923 in the correlation network, compared to 76 in the TE network (summing both in- and out-edges). The high node degree in the correlation network suggests that correlation testing may be overly permissive, with less informative edge weights.

Clauset, Shalizi, and Newman’s method for statistically determining whether a network is ‘scale-free’ showed that the TE network is not [27]. Using the TE network, the result showed alpha = 2.17, which is consistent with power law networks. However, the goodness of fit test using the Kolmogorov-Smirnov statistic produced a p-value of 0.011, indicating that only a small fraction of the simulated scale-free distributions are "close" to the observed degree distribution.

In the rest of the analysis, only the transfer entropy network is used, since it is clear that the correlation-based network is not a super-set of the transfer entropy network, does not agree in the weighting, and is likely overly permissive with regard to active interactions.

### Influence ranking through iteratively solving the influence maximization problem

Using transfer entropy to quantify information flow, if an upstream node transfers information to a downstream node, respecting edge directions, the downstream node is said to be 'influenced'. The area of influence can be found by application of a diffusion process, where the flow follows edges with greater information transfer (edges with greater weights), ‘visiting’ nodes and resulting in a cover on the network. The maximization problem involves finding a set of nodes with size *K*, that when treated as sources, influences the largest proportion of the network, which is to say, that after the diffusion process is applied, no other set would lead to a greater network cover.

The Influence Maximization Problem (IMP) was solved over a range of set sizes, *K* = 1 to 50. Since ant optimization is stochastic and can result in variable solutions, two different parameter sets were used (S1 Text). First a ‘slow’ parameter set was used (best of 8 restarts, 64 ants, 32 local optimization steps, evaporation rate 0.2). The range of *K* was run three times, for a total of 150 ant-optimization runs. A count was made on the number of times genes were selected across solutions. As an example, if a gene appeared in 46 solutions, on average, for *K* = 1 to 50, it would be considered a high-ranking gene. The influence score, representing a network cover, increased quickly for small values of *K*, gradually leveling out. With *K = 44* source nodes (3% of the network), a maximum network cover of 1,308 nodes (93%) was produced. Beyond *K = 44*, the score increased by single digits through the addition of single nodes (see S2 Fig). Regarding the rate of change in network cover, from *K* = 1 to *K* = 2, the total network cover increased 12%. However, after that, the rate of increase drops quickly. Between *K* = 14 to *K* = 15, the network cover increased at a rate of less than 1%, and after *K* = 24, for each additional node added to the set of sources, the increase in network cover dropped to less than 0.5%. The top ranked gene FKH1, was selected on average 49 (out of 50 possible) times, followed by two genes, SFP1 and TFC7, that were selected on average 47 and 46 times respectively. Overall, 52 genes were selected in at least one run.

A second parameter set, the ‘fast’ set, used 4 restarts, 16 ants, 8 local optimization steps, evaporation rate 0.2. For each value of *K*, 49 optimizations were run, for a total of 2,450 result sets. We found that faster optimization runs lead to more variation in the results. However, using the same ranking method, counting the number of times a gene was selected, resulted in excellent agreement with the ‘slow’ parameter set (S1 Text, S3 Fig). The set of genes in the top 15 ranked influencers are identical across parameter sets. The top 15 influencers from both parameter sets are found in Table 1.

**Table 1:** Top 15 ranked influencers and associated centrality metrics.

Gene	Slow Rank	Fast Rank	Alpha Central	Degree	Strength	Authority	Ego2	SubGraph Centrality	Betweenness	1-Constraint	Hub Score
**FKH1**	1	9	4.61	92	53.18	0.148	191	0.00	1005	0.019	0.740
**SFP1**	2	1	1	76	33.88	0	146	143.78	0	0.014	0.003
**TFC7**	3	2	1	140	73.85	0	164	108.33	0	0.008	0.530
**RAP1**	4	3	2.37	87	47.78	0.096	159	0.00	1365	0.019	0.434
**GCN5**	5	4	1	73	46.11	0	175	52.67	0	0.016	0.624
**SOK2**	6	6	1	7	2.93	0	82	4.78	0	0.197	0.000
**RFX1**	7	5	1	58	29.96	0	62	49.33	0	0.019	0.006
**CBF1**	8	12	1	8	3.23	0	104	3.83	0	0.145	0.014
**MED2**	9	7	1	48	23.86	0	58	50.67	0	0.022	0.022
**STP1**	10	9	1	55	26.81	0	64	80.17	0	0.019	0.015
**MBP1**	11	10	1	41	22.01	0	95	202.67	0	0.039	0.285
**YAP1**	12	11	1	43	22.34	0	50	27.00	0	0.025	0.006
**DAL82**	13	14	1	6	3.39	0	46	203.00	0	0.181	0.003
**MED4**	14	13	1.62	103	56.26	0.0002	144	0.00	290	0.011	0.022
**ABF1**	15	15	1	39	19.95	0	70	19.5	0	0.026	0.080

### Comparing influence to more traditional metrics of centrality

To provide a basis for comparison to the ranked influencers, 13 different centrality measures were computed on the TE network. Brief descriptions of each centrality metric can be found in supplementary text (S1 Table).

**Fig 3:**
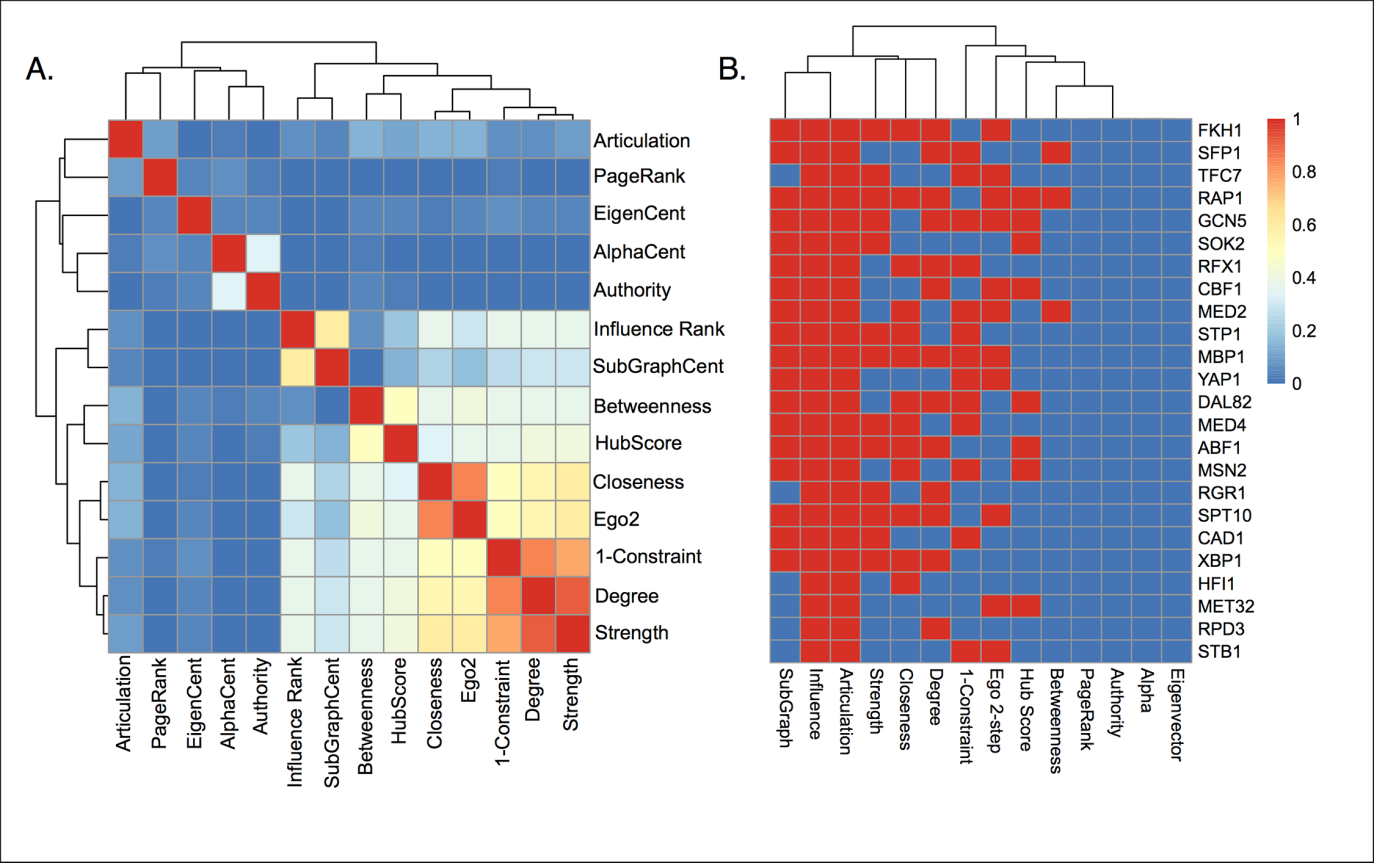
**(A) The Jaccard index was used to compare centrality measures.** The top 24 ranked genes from 14 different centrality measures were compared using the Jaccard index, which gives values of 1.0 for perfect agreement between sets, and 0 for disjoint sets. All genes from the articulation set were used as they have binary values. **(B) Highly influential genes often selected by other centrality metrics.** Genes are sorted by influence ranking in rows (top to bottom), and centrality metrics are found in columns.

As stated earlier, after *K* = 24, the increase on network cover had dropped below 0.5%, making this a reasonable stopping point in selecting the most influential genes. To compare with other metrics, the top 24 genes were selected for each centrality measure. A Jaccard index was computed for each pair of centrality measures (Fig 3), and although some clustering is observed among centrality metrics, especially among node-degree related measures, there remains substantial disagreement in top ranked genes.

The top ranked influential genes are not found among highly ranked genes in eigenvector based centrality measures including authority, eigenvector centrality, and alpha centrality. However, eigenvector related measures of centrality revealed important genes that are not found in other lists. For example, the well-known cell cycle regulatory gene CLB2 was selected by alpha centrality and authority, while it was not found using influence ranking or betweenness. Overall, no ranked list contained a definitive set of cell cycle related regulators. Across measures, gene set enrichment showed a wide variety of associations with biological processes, illustrating differences in the gene rankings (S2 Text).

### Influential topology in the regulatory network

**Fig 4:**
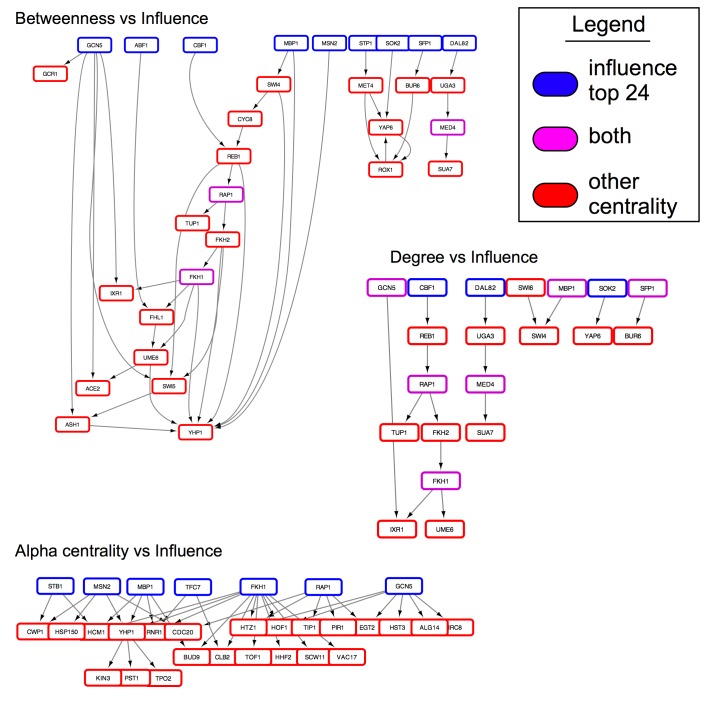
Topology of influential nodes. Highly influential nodes (blue) tend to be upstream of other genes (red) selected by a variety of centrality metrics (edges are directed towards the bottom of the figure). Genes selected by both centrality metrics are shown in purple. For more centrality metrics, please see S4 Fig.

We have found that within the regulatory network structure, the influential genes tend to be situated upstream of genes selected by other centrality measures (Fig 4, S4 Fig).

**Fig 5:**
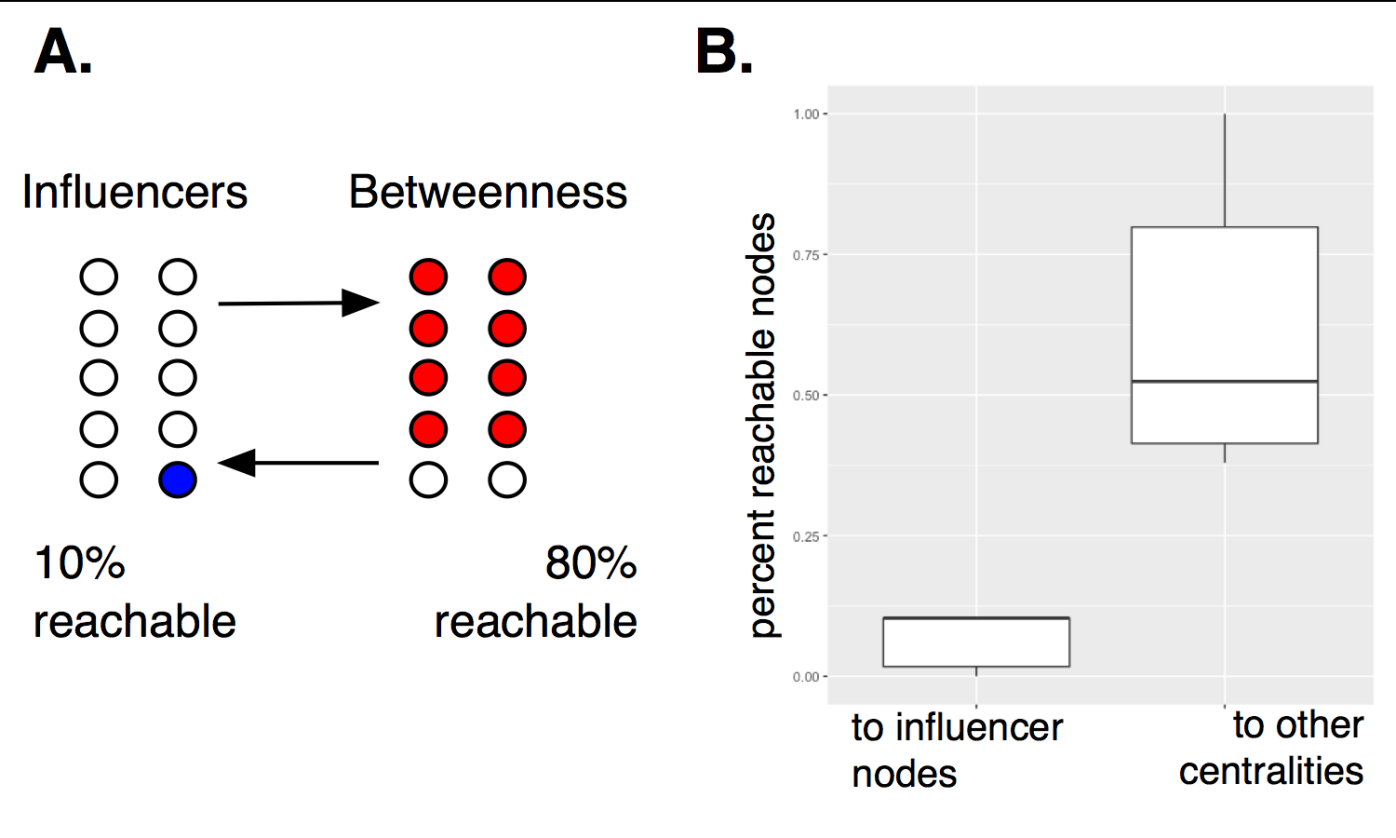
Influence can be quantified by computing node reachability. In (A), an example of node reachability is shown. After starting from a defined set of nodes, *O*, a node, *v*, is considered reachable if there exists a directed path leading from any node in *O* to *v*. For example, starting at the set of influential nodes, 79% of top ranking nodes using the betweenness measure can be reached, compared to only 12% of influential nodes after starting at the “betweenness nodes”. Overlapping nodes found in both sets have been removed. In (B) node reachability over all centrality measures is aggregated in a boxplot.

For example, the influencer genes act as regulators for genes selected by alpha centrality, while no genes selected by alpha centrality regulate the influencer genes. The same is found for the eigenvecteor centrality and betweenness sets. In some cases, there is a fair amount of overlap in the top-level regulators, such as among the high degree nodes and the articulation set. But, overall, we see the influencers stay as top-level regulators to genes selected by other centrality measures. This can be quantified by computing the fraction of reachable genes, starting at a given measure, and excluding overlapping genes (Fig 5).

For example, starting at the set of influential genes, 79% of the betweenness selected genes can be reached, while starting at the betweenness genes, only 12% of influencers can be reached. Starting at the influencer genes, 41% of degree central nodes can be reached, while only 12% of influencers can be reached from the degree central nodes. Starting from every centrality measure, the fraction of reachable nodes is fewer, compared to starting from the influential genes. On average, 54% of “central genes” (excluding subgraph centrality) can be reached when starting at the influential genes, compared to 8% of reachable influential genes, after starting from “central genes” of other measures. Subgraph centrality forms a strong intersection with the influential genes, resulting in no connections between sets. These influential genes are, in a sense, topologically central and connect to important genes found by other centrality measures.

### Evaluation of top ranked genes

Since the yeast cell cycle has been the subject of many studies, we have data and results from other projects which we can use in the evaluation of the algorithm.

First, we examined the experimental outcomes for yeast genetic experiments found in the SGD [28]. In order of influence ranking, large-scale genetic survey phenotypes are listed in Table 2, as well as PubMed Central IDs for papers showing evidence of cell cycle regulation. If a direct cell cycle related phenotype was found, it was reported in Table 2. But given the close connection between lifespan, metabolism and the cell cycle, if no direct cell cycle phenotype was found, then a related phenotype was reported. It should be noted that even MBP1, which is clearly involved in the G1/S transition, does not have a phenotype listed that directly mentions the cell cycle. Nearly all ranked genes have phenotypes that are in some way related to cell cycle, metabolism, or longevity.

**Table 2:** Influential ranked genes show evidence for association to cell cycle.

Influence ranking	Gene	Genetic experiment	Phenotype	Evidence as a cell cycle regulator
1	FKH1	Null	Altered rates of cell cycle progression through the S and G2/M phases	PMC3872199
2	SFP1	Null	Cell cycle progression in G1 phase: delayed, increased duration	PMC1460418
3	TFC7	Null	Inviable	PMID:9584160
4	RAP1	Null	Inviable	PMC1637117
5	GCN5	Null	Chronological lifespan: decreased	PMC3771362
6	SOK2	Classic Over Expr.	Cell cycle progression in G1 phase: abnormal	PMC3872199
7	RFX1	Null	Cell cycle progression in G1 phase: delayed	PMC1291218
8	CBF1	Overexpression	Cell cycle progression: abnormal	PMID:18617996
9	MED2		Transcriptional regulator	
10	STP1	Null	Cell cycle progression in G1 phase: increased duration	PMC2613934
11	MBP1	Null	Cell size: increased	PMID:15965243
12	YAP1	Overexpression	Cell cycle progression: abnormal	(YAP6 has evidence)
13	DAL82		Parallels DNA during cell cycle	PMC4384442
14	MED4	Null	Inviable	
15	ABF1	Null	Inviable; conditional mutants show delayed progression through G2 phase	PMC1637117

To compute gene set enrichment, over-representation testing was performed using the ConsensusPathDB service, which utilizes a hypergeometric test over a large collection of pathways and gene ontology (GO) terms [29]. P-value adjustment is done using FDR correction and a background of 4,766 genes was used relating to the array used. Gene set enrichment showed that the influence ranked genes were significantly associated with cell cycle related pathways and cell cycle related GO categories. The “regulation of transcription involved in G1/S phase of mitotic cell cycle” GO term (GO:0000083) had a q-value of 1.1e-4, the "regulation of transcription involved in G2/M-phase of mitotic cell cycle" GO term (GO:0000117) had a q-value of 1.08e-3 and the cell cycle phase (GO:0022403) had a q-value of 0.008. The KEGG pathway “Cell cycle—yeast—Saccharomyces cerevisiae (budding yeast)” had a q-value of 0.02.

**Fig 6:**
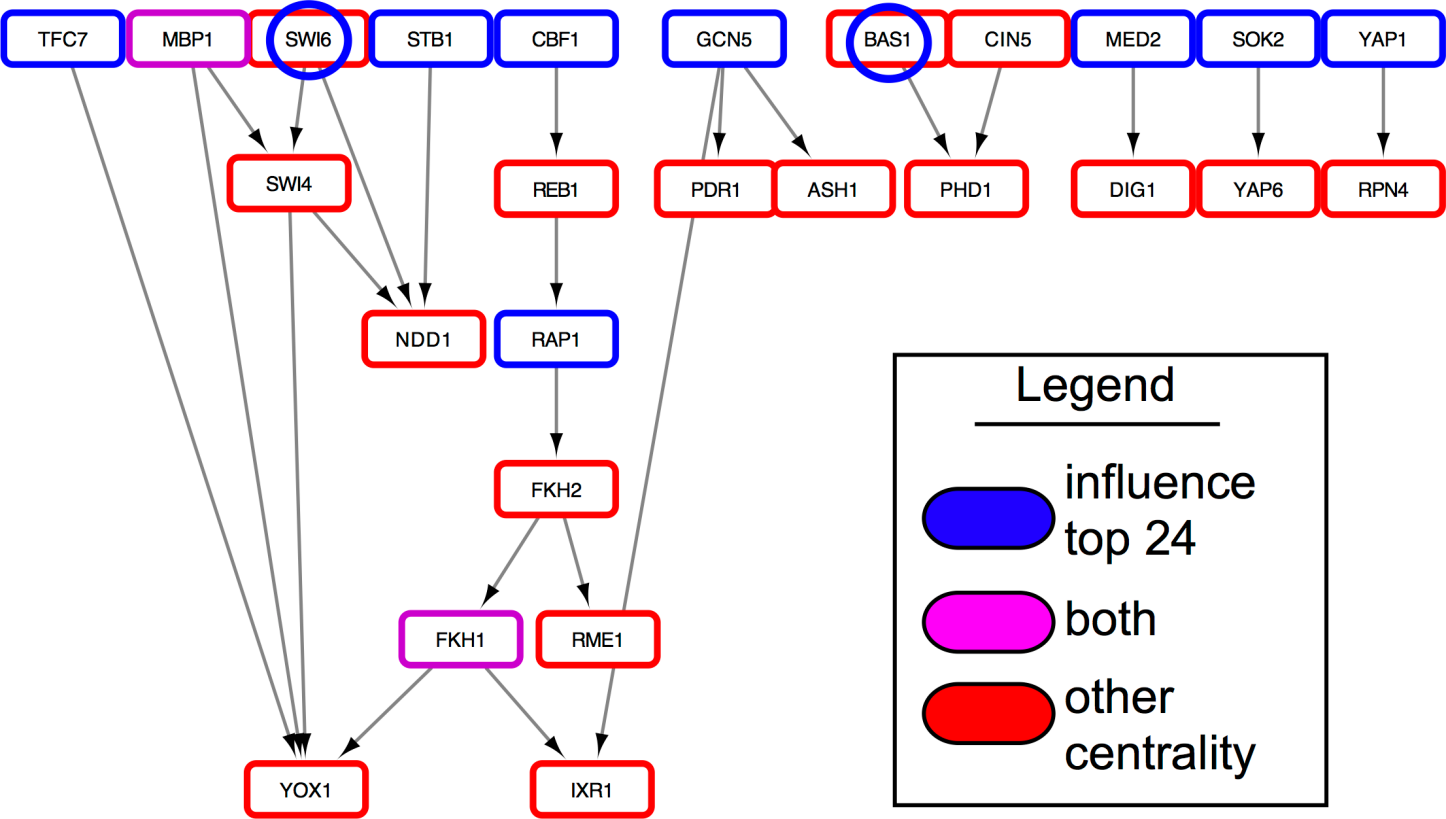
Influence ranking of cell cycle related transcription factors. Of the 32 cell cycle related transcription factors given by Eser et al. [5] (in red), most are directly downstream of influential genes (blue). Purple shows an overlap between influential and Eser selected genes and blue-circled genes show low ranking influential members.

In Eser et al., the source of the data, 32 hypothesized cell cycle regulators were named [5], five of which are found in the 24 top ranked influencer list. Comparing the top ranked influential genes, we see again that the influential genes are immediately upstream of the Eser TFs (Fig 6), where in total, out of 27 TFs in the network, 15 cell cycle regulators overlap with the influential list, or are regulated by influential genes. Two more, SWI6 and BAS1 were selected as low ranking influential genes (ranks 33 & 34). Therefore, the influential ranked list contained or regulated 63% of the available Eser genes.

Recently a cell cycle model by Tyson et al. that successfully accounts for 257 of 263 phenotypes [30] was published. In total, 29 genes were extracted from the model where complexed genes were considered separately (e.g. SWI6 and SWI4 were used instead of SBF). The full YeastMine network scaffold contained 28 of the 29 genes (CDC55 was not present), and 20 genes were in the TE network. Three genes from the model were ranked as influencers (MBP1, SWI4 and SWI6).

While most of the Tyson model genes are not ranked influencers, they are immediately regulated by influential genes. SWE1 is regulated by 4 ranked genes. CDC20 is regulated by 2 ranked genes. CLB5 is regulated by 2 ranked genes. SIC1 is regulated by 1 ranked gene. So, in almost all cases, the Tyson model genes are not regulated by a single influencer, but by multiple influencers. This shows that even though the mechanistic modelers have different goals–the derivation of small models consisting of well-known elements on multiple levels (protein level and others) that produce a desired behavior, such as cell cycle timing, and timing changes with given mutations–there is a clear relation to the influential genes.

## Discussion

Transfer entropy has been shown to be useful in quantifying information transfer. Here, we showed that using time lagged transfer entropy, along with a permutation testing framework, leads to biologically salient network structures. Even though the network was constructed by considering all possible regulatory edges, it recovers much of the structure and functional enrichment that one would expect, as demonstrated by the lists of genes returned by commonly used centrality metrics, such as betweenness and degree.

Edges with the highest weights, implying greatest information transfer, include (SWI4 → SPT21, TE = 1.57), (TFC7 → MSL1, TE = 1.36), (FKH2 → ALK1, TE = 1.34), (TFC7 → CHL1, TE = 1.27) and (SWI4 → RNR1, TE = 1.27). The source nodes are well-known, multi-functional transcription factors, while the target nodes have more focused functions. SPT21 has a role in regulating transcription through chromatin silencing. MSL1 is involved in mRNA splicing through interactions with the U2 small nuclear RNA. ALK1 is involved in proper spindle positioning and nuclear segregation following mitotic arrest. CHL1 is related to the cohesion of sister chromatids during mitosis. Finally, RNR1 plays an essential role in the cell cycle, assisting with DNA replication and repair. More well-known cell cycle interactions also have high TE edge weights. These include SWI4-SWE1 (TE ranked 7th highest out of 2,084), NDD1-SWI5 (ranked 17/2084), RAP1-FKH2 (ranked 20/2084), and SWI4-YHP1 (ranked 30 / 2084).

Yeast is often used as a model organism in the study of aging. Interestingly, the top two most influential genes, FKH1 and SFP1 have both been related to lifespan [31–34]. The close ties of sources and edge weights to the cell cycle process show that the general dynamics of the cell cycle were captured, reinforcing the usefulness of transfer entropy in biological investigations.

Some well-known cell cycle regulators, such as NDD1, were not selected by influence maximization. In cases such as this, it can often be explained by exploring the immediate neighborhood. In the TE network, NDD1 has upstream regulators FHL1, STB1, SWI4 and SWI6 (three of which are ranked influencers). NDD1 itself targets 18 other genes, all with no influence ranking. Among the targets, we found ALK1, which is also a target from FKH2 as mentioned earlier, as well as CLN1, which is also targeted by three influencers FKH2, SWI4, and SWI6. So, although NDD1 is famous as a cell cycle regulator, when solving the IMP, there are more optimal sources that target the same downstream genes.

When we considered the ranking of influential genes, we saw that high-ranking genes were also more likely to be ranked by other centrality metrics. But there are several notable exceptions. SWI4 and SWI6 were relatively low ranked influencers, but were highly ranked by other metrics. These examples are notable due to their established role in the cell cycle and regular inclusion in models. Proteins SWI4 and SWI6 are members of the SBF complex, interacting with the MBF complex (SWI6-MBP1) to regulate late G1 events. The “low” influence ranking was due to higher ranked influencers being upstream in the regulatory network. Therefore, they were only selected as *K*, the set of requested influencers, grew large enough.

Network control is one goal in the study of dynamic networks [35,36]. Given that influential nodes seem to have a topologically advantageous position, one could speculate that influential genes might be useful selections for network control. Biological events that impact the influential nodes, thereby affecting normal information flow, could have a strong effect on the network, potentially leading to disease states. Discovering the minimum sets of biological entities that hold the greatest influence in the network context could lead to further understanding of how network dynamics is associated with disease.

## Materials and methods

The work in this paper can be summarized in a few important steps that are discussed in more detail below: 1) time lagged variants of Spearman's correlation and transfer entropy are described, which were used in constructing the genetic regulatory network; 2) the diffusion model is described, which forms the basis of the score function; and 3) the ant optimization method is described, which was used to maximize the score function, thereby solving the IMP.

The methods described here have been implemented in python and are freely available. Run times are kept low by computing the diffusion using sparse matrix linear solvers, and using a multicore-parallel strategy for performing ant optimization. The network weighting, optimization, and diffusion methods are independent, allowing researchers to "mix-and-match" their favorite modules.

### Data sources

Eser et al. [5] generated time series expression data from two replicates of synchronized yeast producing metabolically labeled RNA levels every five minutes over 41 time points. The expression series spans three cell cycles, which progressively dampen in wave amplitude, as yeast synchrony is lost. Using a model for detecting periodicity in gene expression, 479 genes were labeled as statistically periodic. Additionally, 32 transcription factors were predicted to be cell cycle regulators.

YeastMine, the database of genetic regulatory interactions in yeast (May 2015) [4] provided regulatory edges. Using 6,417 yeast genes, 26,827 genetic regulatory edges were collected. Edge weights were computed using a variation of transfer entropy, as described below.

The Saccharomyces Genome Database (SGD) was used to reference experimental phenotypes and gene annotations [28].

### Computing weights with transfer entropy and time lagged Spearman's correlation

Given two genes connected by an edge, the edge weight was computed in two ways. First, time lagged Spearman's correlation was used with time lags of 0 to 5 steps (0 to 25 mins.), keeping the maximum. Second, time lagged transfer entropy (TE) was used, similar to what is described in [37,38]. TE is computed at each time lag along with a robust distance comparing the observed TE to TEs generated from permuted data. The TE and lag time is returned that maximizes this distance.

Time lagged Spearman's correlation is computed by taking two time series, or numeric vectors ***x*** = {*x*_1_,*x*_2_,…,*x*_*n*_} and ***y*** = {*y*_1_,*y*_2_,…,*y*_*n*_}, and computing the correlation on sub- sequences {*x*_1+*k*_,…*x*_*n*−1_,*x*_*n*_} and {*y*_1_ *y*_2_,…*y*_*n*−*k*_}, where *k* is some integer representing the time lag between variables.

Transfer entropy (TE) is an information theoretic quantity that uses sequence or time series data to measure the magnitude of information transfer between variables [25,38]. Transfer entropy is model-free, directional, and shown to be related to Granger causality [39]. In TE, given two random variables variables *X* and *Y*, where *X* is directionally connected to *Y* (or *X* → *Y*), we would like to know if prior states *X* help in the prediction of *Y*, beyond knowing the prior states of *Y*.

Given two sequences ***x*** and ***y***, we describe transfer entropy as

$$T_{x\to y}^{\left(k\right)}=\sum_{y_{t},y_{t-1},x_{t-k}}{P(y}_{t},y_{t-1},x_{t-k})\;\log\frac{P(y_{t},y_{t-1},x_{t-k})P(y_{t-1})}{P(y_{t-1},x_{t-k})P(y_{t},y_{t-1})},$$

where ***x***_***t−k***_ indicates value of the sequence at time step *t* − *k*.

To perform the computation, first ***x*** and ***y*** are mean-centered and scaled to be within the range [−1,1]. A Gaussian kernel density estimate (KDE) is fit with a bandwidth given by “Scott’s rule”. Then, a three-dimensional grid is generated by equally spacing some number of points between −1 and 1 in each dimension. Using the grid, points are sampled from the KDE, creating a joint probability distribution, which is normalized in order to sum to 1. The required distributions are marginalized from the joint distribution by summing across the grid. Smaller grid sizes provide a finer grained probability distribution, but slow the computation without changing the values substantially. A three-dimensional grid of *10*^*3*^ points was found to be a good compromise between computation time and accuracy.

A permutation test was performed to assess statistical significance of the transfer entropy, *T*_***x***→***y***_. The sequence ***x*** was split into a list of subsequences with length 3 and permuted 50,000 times. A robust distance, $\left(T_{x\to y}-Median(T_{x\to y}^{perm}))/MAD(T_{x\to y}^{perm}\right)$, was computed where *T*_***x***→***y***_ is the observed transfer entropy and $T_{x\to y}^{perm}$ is the set of TEs resulting from permuted sequences, and the ***MAD*** is the median absolute deviation. The time lag maximizing the robust distance is selected and a p-value is computed by taking a count on the number of times the permuted TE was greater than the observed TE, giving an empirical p-value. Edges were accepted if empirical p-values were less than or equal to 1/(*p*_*n*_ + 1), where *p*_*n*_ is the number of permutations (*p*_*n*_ = 50,000).

### The diffusion model is used to score solutions to the IMP

The IMP maximizes a network cover based on diffusion. The diffusion model, and most of the nomenclature, is described in [15]. The diffusion models are Markov chains with absorbing states [40]. In the model, vertices are first partitioned into sets S ⊆ V and T ⊆ V, where V is the set of all vertices. The set S contains sources, which in the model are generating information flowing through the rest of the network (nodes in T) until reaching a dead end or absorbing back into S.

The stochastic matrix, defining the probability of moving from one vertex to another, is defined as

$$p_{ij}=\frac{w_{ij}}{\sum_{j}w_{ij}},$$

where edge weights *w*_*ij*_ are the weights on outgoing edges. Sets S and T partition the stochastic matrix as

$$P=\left[\begin{array}{cc} P_{SS} & P_{ST}\\ P_{TS} & P_{TT} \end{array}\right],$$

where **P**_SS_ defines the transition probabilities from nodes in S to S, and **P**_ST_ defines transition probabilities from S to T, and so on. Although the matrix is square, it is not symmetric, given the directed edges.

Ultimately, we wish to compute the expected number of visits from a node *v*_*i*_ ∈ S, to a node *v*_*j*_ ∈ T, defined as matrix **H**. At time step *t*, information can travel from *v*_*i*_ ∈ S to *v*_*j*_ ∈ T directly, or it would already be at adjacent node *v*_*k*_, and would travel from *v*_*k*_ ∈ T to *v*_*j*_ ∈ T in the next time step. So, at time point *t*, the estimated number of visits from *v*_*i*_ ∈ S to *v*_*j*_ ∈ T is given as

$$h_{ij}^{\left(t\right)}=p_{ij}+\sum_{k\in T}h_{ik}^{\left(t-1\right)}p_{kj},$$

where *p*_*ij*_ is the transition probability of *v*_*i*_ ∈ S to *v*_*j*_ ∈ T, $h_{ik}^{\left(t-1\right)}$ is the expected number of visits that have already taken place at time (*t* − 1), from *v*_*i*_ ∈ S to *v*_*k*_ ∈ T, and *p*_*kj*_ is the probability of the transition from *v*_*k*_ ∈ T to *v*_*j*_ ∈ T. The matrix form of the equation is

$$H^{\left(t\right)}=P_{ST}+H^{\left(t-1\right)}P_{TT}.$$

In the long run, at steady state, when **H**^(*t*)^ ∼ **H**^(*t*−1)^, the equation reduces to **H**(**I** − **P**_TT_) = **P**_ST_, where **I** is the identity matrix. By taking the transpose of both sides, we have ${\left(I-P_{TT}\right)}^{\prime }H^{\prime }=P_{ST}^{\prime }$. This form lets us avoid the matrix inverse when solving for **H**, which can be expensive or impossible to compute given that the directed network is represented as an asymmetric matrix. Fortunately, the appropriate iterative solvers are available in the Python SciPy sparse linear algebra library and are robust enough to handle singular matrices.

To compute a measure of influence on the network, after solving for **H** the expected number of visits on nodes, the influence is summarized as the “influence-score”,

$$\Omega_{s}=\sum_{i\in S}\left\{\sum_{j\in T}Ι(h_{ij}>\theta )\right\}$$

where h_*ij*_ is the number of visitations (using matrix **H**) from node *v*_*i*_ ∈ S to connected nodes *v*_*j*_ ∈ T. Indicator function I (h_*ij*_ > θ) is equal to 1 if the number visitations is greater than a threshold *θ*. The sum of edge weights, ∑_*i*∈*S*_ *w*_*i*_, is used as a tie-breaker in the case of degenerate solutions. Degenerate solutions refer to the situation where different solution sets produce an identical cover on the network. In that case, we would like to give preference to the solution that contains nodes with higher overall edge weights, indicating greater degree of information transfer to the network, and potentially greater influence. This influence score is equivalent to computing the cover on nodes in T. In this work, *θ* = 0.0001 is used, which was selected after observing values in H.

### Ant optimization is used to search for influential nodes

An implementation of the hypercube min-max ant optimization algorithm was used to search for solutions to the Influence Maximization Problem [41,42]. Ant optimization is based on the idea of probabilistically constructing potential solutions to a given problem, in this case a subset selection problem, and reinforcing good solutions with a "pheromone" weight deposited on solution components, ensuring that good solutions become increasingly likely in later iterations.

Since the algorithm is stochastic, and results can vary, the optimization is repeated for a defined number of runs. The main results were produced using a ‘slow’ parameter set, using 8 restarts per value of *K*, 64 ants, and 16 local optimization steps (full parameterization is given in S1 Text). Each convergence (before restarting) takes a number of iterations where ants construct solutions, perform a local search, score the solutions using the influence score, and reinforce the components in that order. As a run progresses, the pheromone values move to either one or zero, indicating whether the component was selected. The goal of the optimization is to find the subset S ⊆ V of vertices such that

$$S_{opt}={\mathrm{argmax}}_{\left\{S\subseteq V:|S|=K\right\}}\Omega_{s}.$$

At the start of each iteration, ants construct potential solutions, a subset of vertices, by sampling from nodes using probability distribution

$$q_{i}=\frac{u_{i}^{\alpha }r_{i}^{\beta }}{\sum u_{i}^{\alpha }r_{i}^{\beta }},$$

where *q*_*i*_ is the probability for sampling any node *v*_*i*_, with the sum of outgoing edges giving node weight *u*_*i*_ and pheromone weight *r*_*i*_. The *α* and *β* parameters are used to give importance to either node weights or pheromones. Solutions are constructed by sampling one node at a time. After each sample, the probabilities are renormalized. Here, *α* and *β* are set to 1.

Local search is performed by stochastic hill climbing, where we try alternative solutions produced by random single bit flips. If a better score is found, the solution is replaced, and carried forward. Local search has a fairly strong effect on the quality of the solutions, and even a small number of hill climbing steps tends reduce the time required for convergence.

Next, using the influence score function, each potential solution is scored, with the best solution kept and compared to solutions found in earlier runs. As part of the Min-Max algorithm, three solutions are kept throughout the run: the iteration-best, the restart-best and the overall-best. The pheromone updates use a weighted average over the three solutions. At the beginning of the run, the pheromone updates are entirely from the iteration-best solution, but gradually, the updates are increasingly influenced by the restart and overall-best solutions, which is done to avoid local minima. The weighted average pheromone would be *r*_*avg*_ = *f*_1_*b*_*i*_ + *f*_2_*b*_*r*_ + *f*_3_*b*_*b*_ where *b*_*i*_ is the iteration best, *b*_*r*_ is the restart best, *b*_*b*_ is the best overall, and fractions *f*_1_ + *f*_2_ + *f*_3_ = 1. The pheromone updates are defined as *r*^(*t*+1)^ = *r*^(*t*)^ + *d* (*r*_*avg*_ − *r*^(*t*)^), where *r*^(*t*)^ is the pheromone weights at time *t*, *d* is the learning rate, and *r*_*avg*_ is the average over the three solutions. Eventually, the pheromone weights become sufficiently close to zero or one, and the rate of change among the weights slows. When the difference in sums over the last solution (all *r*) and the next solution is less than 0.0001, the solution is returned along with the influence score.

### Additional ‘off-the-shelf’ analysis

BioFabric, R and the R packages igraph, pheatmap and ggplot2 were used for visualization and analysis [43,44,45,46]. Cytoscape 3.5.1 was used for visualizing graphs [47,48]. Pathway and GO term enrichment was generated using the CPDB from The Max Planck Institute for Molecular Genetics [49]. SciPy was used in the software implementation [50].

## Supporting information

S1 Fig Similarity metrics vary with the amount of time lag. A.) The transcription factor REB1 interacts with MDH2. Expression levels are shown across three cell cycles where time points are in 5 minute increments. B.) In this example, when time lags are introduced the Spearman's correlation between the two genes decreases. Transfer entropy values show a peak at a time lag of 3.
(TIF)

S2 Fig The network cover, related to *Ω*_*s*_, increases with the number of source nodes (*K*). A highly ranked node will appear in solutions for all values of *K*.
(TIFF)

S3 Fig (A) Average Jaccard across reps. For each value of K, 49 fast runs were performed. Each point represents the mean Jaccard for pairwise comparisons across reps, within a given value of K (x-axis). We see that at smaller values of K, the fast settings return consistent results, while beyond a certain threshold (K = 9), the similarity drops and becomes more unstable. (B) Comparison of influence rankings between fast and slow parameter settings.
(TIF)

S4 Fig Topology of influential nodes in remainder of centality metrics. Highly influential nodes (blue) tend to be upstream of other genes (red) selected by a variety of centrality metrics (edges are directed towards the bottom of the figure). Genes selected by both centrality metrics are shown in purple.
(PDF)

S1 Table Description of centrality metrics. Brief descriptions of the 14 centrality metrics as discussed in the manuscript.
(DOCX)

S2 Table Brief listing of enrichment results using the top 24 influential genes. (DOCX)

S1 Text The ‘fast’ parameter set for ant optimization compares favorably to results from the ‘slow’ parameter set. (DOCX)

S2 Text Functional enrichment on selected genes. To determine gene set functional enrichment, over-representation testing was performed using the ConsensusPathDB service utilizing a hypergeometric test over a large collection of pathways and gene ontology (GO) terms.
(DOCX)

S1 Dataset Four result files are included. time_lag_TE_filtered_edges.tsv—the TF network with TE weights.fast_vs_slow_rankings.txt—the ranking of TFs using both parameter setstop_24_centrality_metrics.xlsx—the centrality metrics for top 24 ranked TFstop_24_ranked_genes_in_each_centrality.tsv—top genes per metric.
(GZ) Click here for additional data file.
