# Correction: Solving the influence maximization problem reveals regulatory organization of the yeast cell cycle

**DOI:** 10.1371/journal.pcbi.1006190

**Published:** 2018-05-23

**Authors:** The PLOS Computational Biology Staff

## Notice of Republication

This article was republished on January 25, 2018, to correct an error in denoting the corresponding author that was introduced during the typesetting process. The publisher apologizes for the error. Please download this article again to view the correct version. The originally published, uncorrected article and the republished, corrected articles are provided here for reference.

## Supporting information

S1 File Originally published, uncorrected article. (PDF)

S2 File Republished, corrected article. (PDF)
