# Supplementary figures and images for: Solving the influence maximization problem reveals regulatory organization of the yeast cell cycle
Source: PLoS Comput Biol. 2017 Jun 19;13(6):e1005591. doi: 10.1371/journal.pcbi.1005591 (PMC5495484; doi:10.1371/journal.pcbi.1005591)

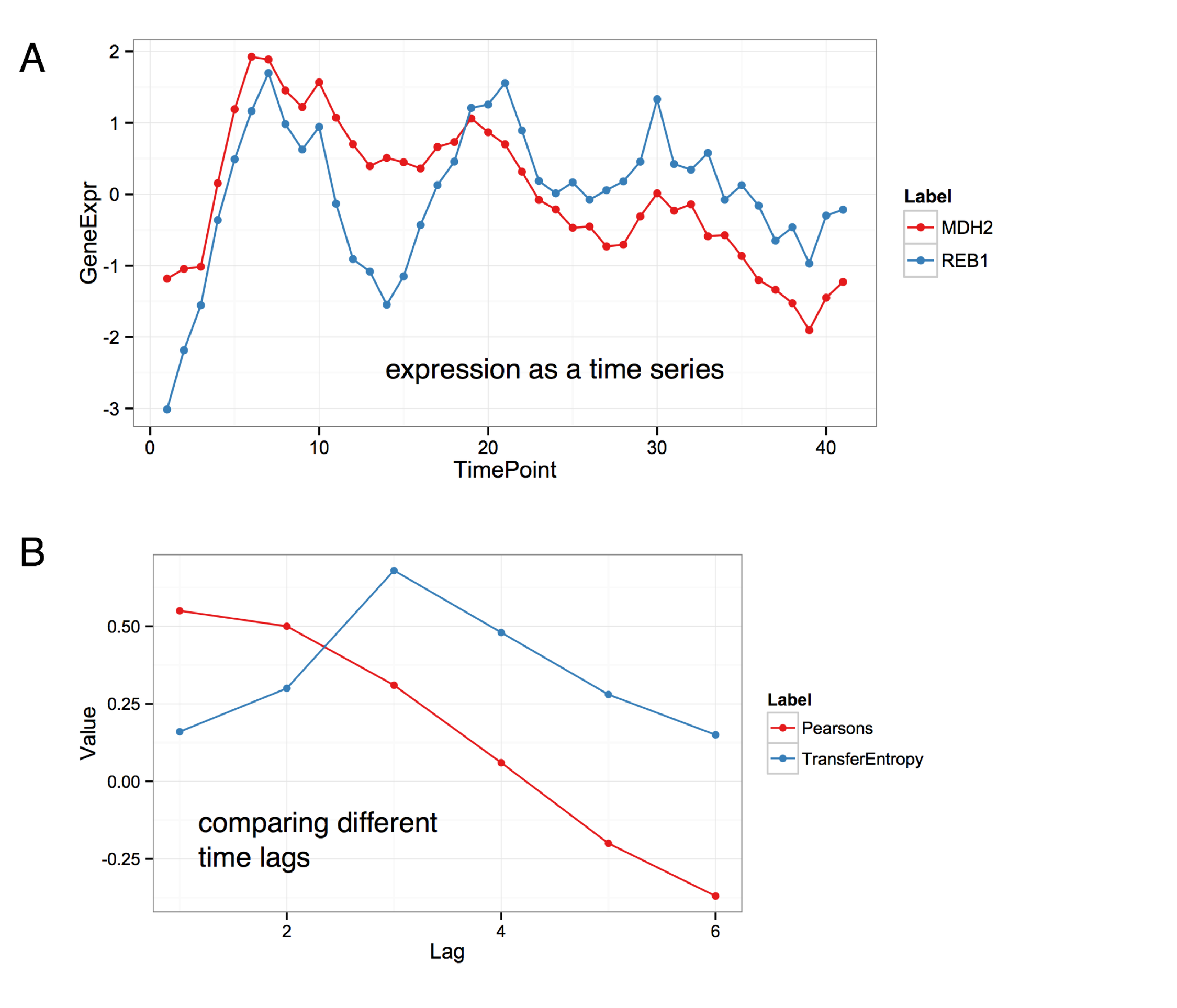

Supplement: S1 Fig — A.) The transcription factor REB1 interacts with MDH2. Expression levels are shown across three cell cycles where time points are in 5 minute increments. B.) In this example, when time lags are introduced the Spearman's correlation between the two genes decreases. Transfer entropy values show a peak at a time lag of 3. (TIF) [file pcbi.1005591.s001.tif]

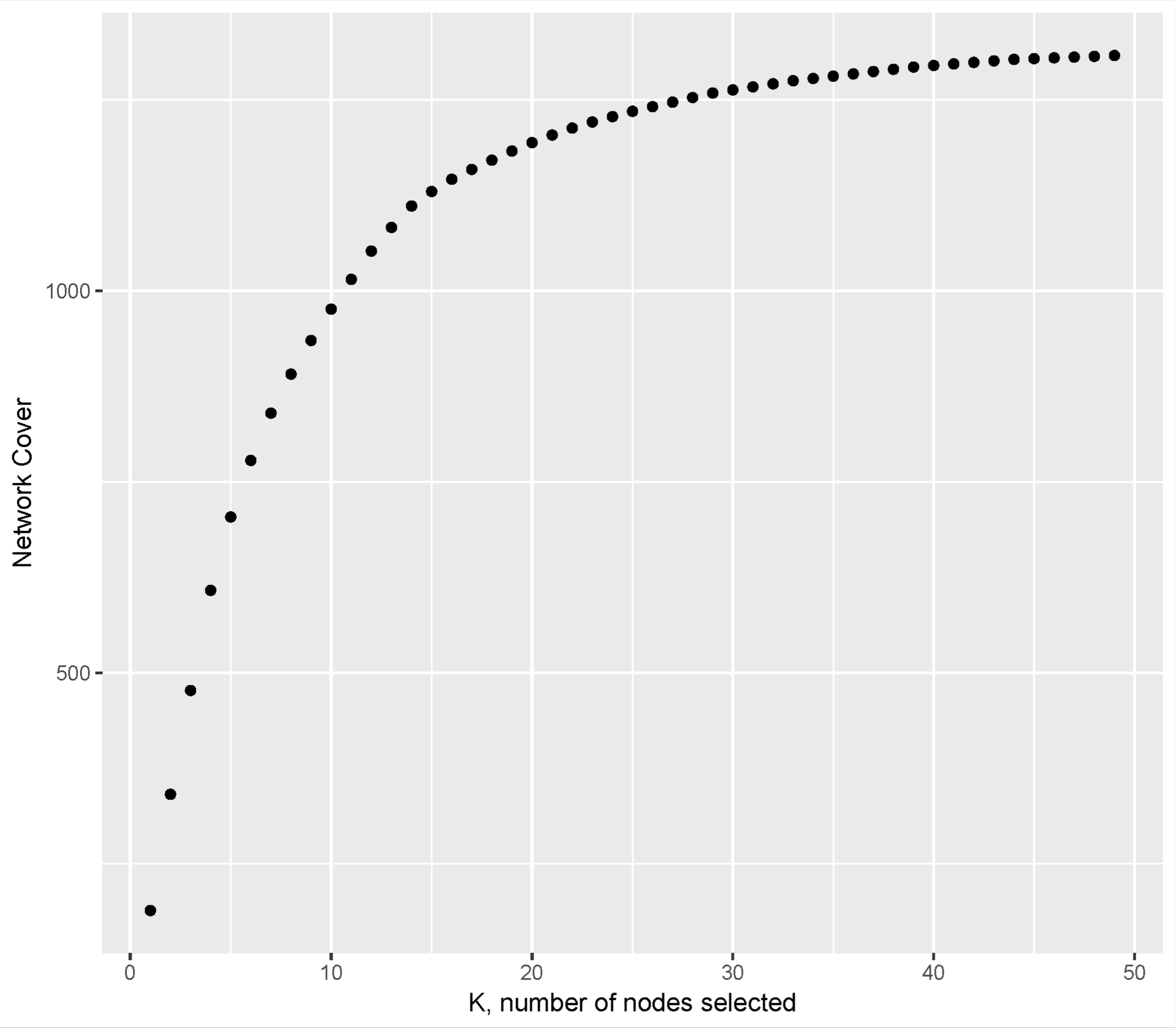

Supplement: S2 Fig — A highly ranked node will appear in solutions for all values of K. (TIFF) [file pcbi.1005591.s002.tiff]

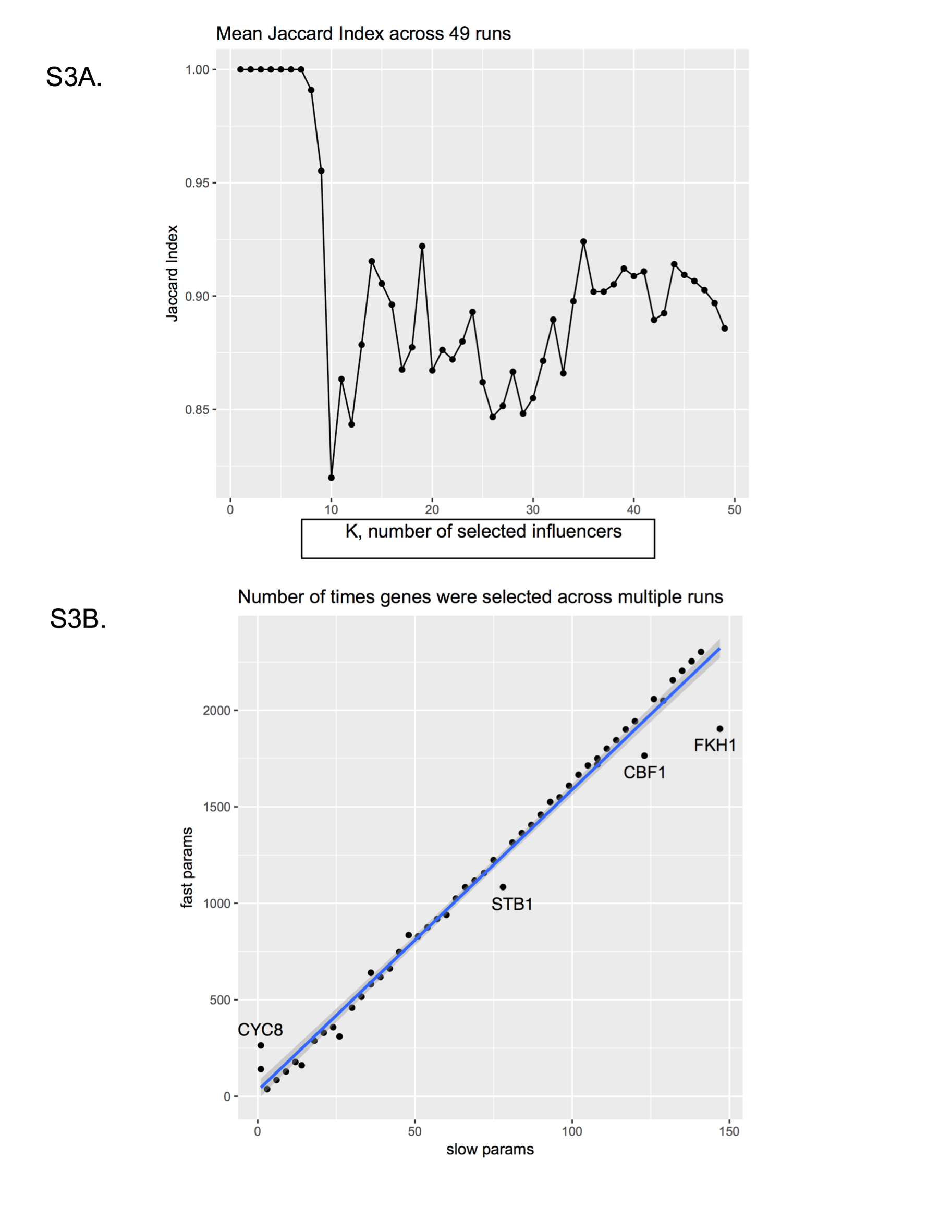

Supplement: S3 Fig — (A) Average Jaccard across reps. For each value of K, 49 fast runs were performed. Each point represents the mean Jaccard for pairwise comparisons across reps, within a given value of K (x-axis). We see that at smaller values of K, the fast settings return consistent results, while beyond a certain threshold (K = 9), the similarity drops and becomes more unstable. (B) Comparison of influence rankings between fast and slow parameter settings. (TIF) [file pcbi.1005591.s003.tif]
