# Supplementary material for: Solving the influence maximization problem reveals regulatory organization of the yeast cell cycle
Source: PLoS Comput Biol. 2017 Jun 19;13(6):e1005591. doi: 10.1371/journal.pcbi.1005591 (PMC5495484; doi:10.1371/journal.pcbi.1005591)

Sub-graphs showing the relationship between influence and various centralities.

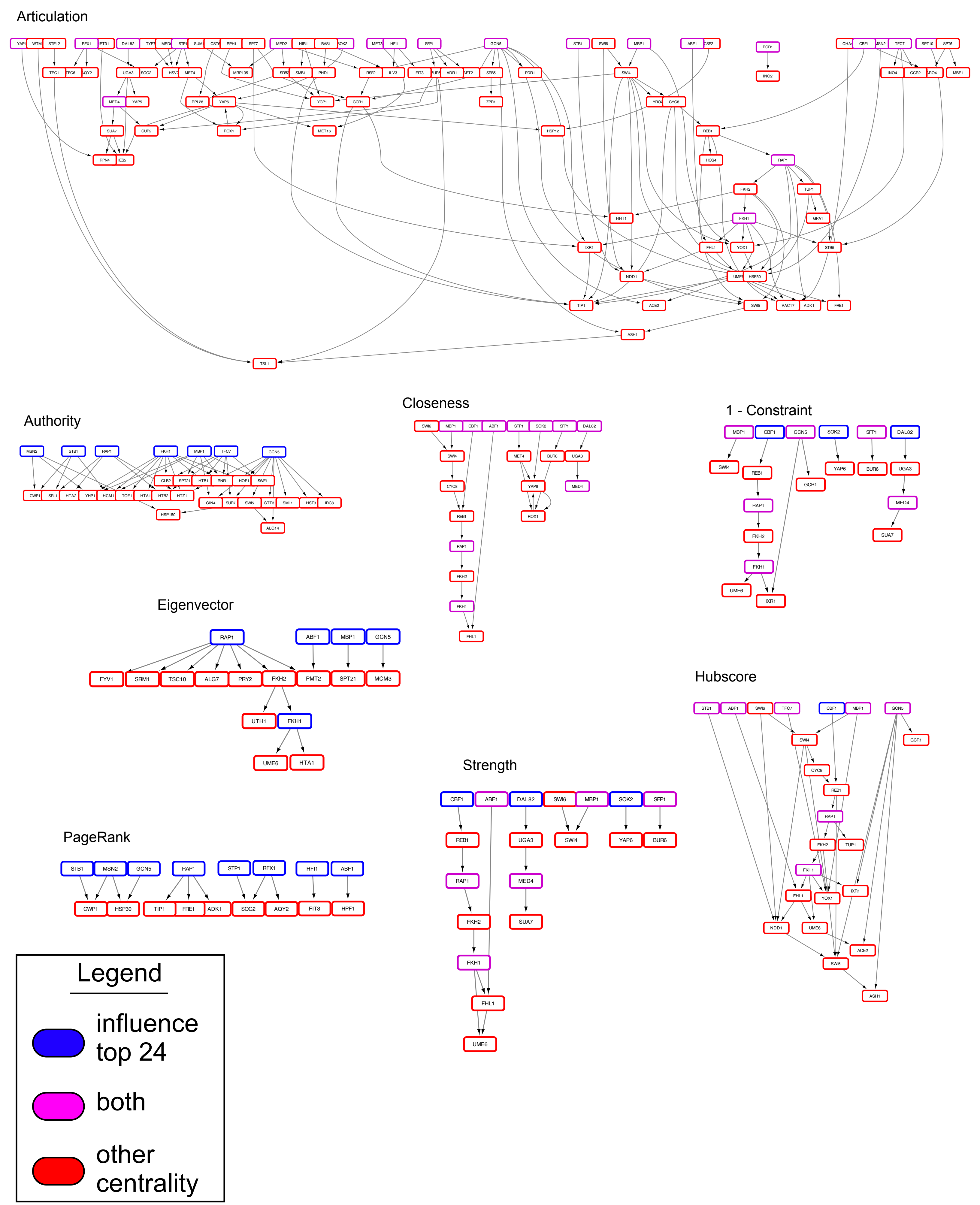

Supplement: S4 Fig — Highly influential nodes (blue) tend to be upstream of other genes (red) selected by a variety of centrality metrics (edges are directed towards the bottom of the figure). Genes selected by both centrality metrics are shown in purple. (PDF) [file pcbi.1005591.s004.pdf]
