# Supplementary material for: Solving the influence maximization problem reveals regulatory organization of the yeast cell cycle
Source: PLoS Comput Biol. 2017 Jun 19;13(6):e1005591. doi: 10.1371/journal.pcbi.1005591 (PMC5495484; doi:10.1371/journal.pcbi.1005591)
Supplement: S1 Table — Brief descriptions of the 14 centrality metrics as discussed in the manuscript. (DOCX) [file pcbi.1005591.s005.docx]

S1 Table. Description of centrality metrics

| Tool | Centrality | | Notes |  |
| --- | --- | --- | --- | --- |
| igraph | Alpha Centrality | Generalization of eigen-centrality for weighted directed graphs. | |  |
|  | Articulation | Also called cut vertices, removal creates separate components. | |  |
|  | Authority | Kleinberg's centrality scores, based on principal eigenvector | |  |
|  | Betweenness | Uses directed and weighted edges to sum shortest paths through a vector. | |  |
|  | Closeness | Inverse of the average length of shortest paths to a vertex in the graph. | |  |
|  | Degree | Sum of edges for each vertex. | |  |
|  | Hub Score | Kleinberg's hub centrality scores, eigenvector based score. | |  |
|  | Ego, 2 steps | Size of the neighborhood two steps out. | |  |
|  | Eigen Centrality | Eigenvector centrality on the undirected graph using weights. | |  |
|  | Page Rank | Google Page Rank, using directed and weighted edges. | |  |
|  | Strength | Sum of edge weights. | |  |
|  | Unconstraint | Equal to (1 – Constraint), using Burt’s constraint method | |  |
|  | SubGraph Centrality | Participation of each node in all subgraphs | | . |
| python | Influence Ranking | Ranked using influence maximization algorithm as described. | |  |
|  |  |  | |  |
|  |  | |  |  |
|  |  | |  |  |
