# Supplementary material for: Solving the influence maximization problem reveals regulatory organization of the yeast cell cycle
Source: PLoS Comput Biol. 2017 Jun 19;13(6):e1005591. doi: 10.1371/journal.pcbi.1005591 (PMC5495484; doi:10.1371/journal.pcbi.1005591)
Supplement: S2 Table — (DOCX) [file pcbi.1005591.s006.docx]

S2 Table. Pathway and GO term enrichment scores

| **Influence ranked genes pathway enrichment** | | | |  |
| --- | --- | --- | --- | --- |
| p-value | q-value | source |  | pathway |
| 0.02 | 0.02 | KEGG |  | Cell cycle - yeast - Saccharomyces cerevisiae (budding yeast) |
| 0.02 | 0.02 | Wikipathways | | Meiosis - yeast - Saccharomyces cerevisiae (budding yeast) |
|  |  |  |  |  |
| **Influence Ranked genes GO term enrichment** | | | |  |
| p-value | q-value | term_goid | term_level | term_name |
| 1.19e-19 | 6.68e-18 | GO:0051171 | 4 | regulation of nitrogen compound metabolic process |
| 5.2e-19 | 1.46e-17 | GO:0009889 | 4 | regulation of biosynthetic process |
| 8.74e-19 | 1.63e-17 | GO:0034654 | 4 | nucleobase-containing compound biosynthetic process |
| 4.57e-18 | 6.03e-17 | GO:0019438 | 4 | aromatic compound biosynthetic process |
| 6.61e-18 | 6.03e-17 | GO:0060255 | 4 | regulation of macromolecule metabolic process |
