# Supplementary material for: Solving the influence maximization problem reveals regulatory organization of the yeast cell cycle
Source: PLoS Comput Biol. 2017 Jun 19;13(6):e1005591. doi: 10.1371/journal.pcbi.1005591 (PMC5495484; doi:10.1371/journal.pcbi.1005591)
Supplement: S1 Text — (DOCX) [file pcbi.1005591.s007.docx]

**SI Text 3**

The ‘fast’ parameter set for ant optimization compares favorably to results from the ‘slow’ parameter set. The parameter differences between the two sets are (slow, fast): restarts (8 ,4), local optimization steps (32,8), the number of ants (64,24).

Fast Parameter Set

number of restarts : 4

number of ants : 16

converge threshold : 0.0001

local optimization : 8

evaporation rate : 0.2

alpha : 1.0

beta : 1.0

mode : tx

optimize on : score

transmit threshold : 0.0001

receive threshold : 0.0001

k : 2

cpus : 4

Slow Parameter Set

number of restarts : 8

number of ants : 64

converge threshold : 0.0001

local optimization : 32

evaporation rate : 0.2

alpha : 1.0

beta : 1.0

mode : tx

optimize on : score

transmit threshold : 0.0001

receive threshold : 0.0001

k : 2

cpus : 4

In the ‘slow’ parameter set, three sweeps of K were performed where K = 1 to 50. Using the ‘fast’ parameter set, 49 sweeps of K were performed. There was excellent agreement among the three slow runs. In the slow runs, 52 genes were selected over all, where the minimum possible would be 50.

In the fast run results, the results variability was higher. When K was greater than 7 (meaning a solution set of 7 nodes), solutions started to vary. The minimum Jaccard index was 0.81. However, in comparing the two parameter sets using the same ranking method, we found that the top 49 genes had excellent agreement in ranking. Of the top 10 influential genes by rank, 9/10 are also in the fast parameter top 10 genes, and 18/20 of the slow influential genes are in the top 20 fast influential genes. See below for a comparison of the top 49 genes between the two parameter sets.
