# Supplementary material for: Solving the influence maximization problem reveals regulatory organization of the yeast cell cycle
Source: PLoS Comput Biol. 2017 Jun 19;13(6):e1005591. doi: 10.1371/journal.pcbi.1005591 (PMC5495484; doi:10.1371/journal.pcbi.1005591)
Supplement: S2 Text — To determine gene set functional enrichment, over-representation testing was performed using the ConsensusPathDB service utilizing a hypergeometric test over a large collection of pathways and gene ontology (GO) terms. (DOCX) [file pcbi.1005591.s008.docx]

**S2 Text. Functional enrichment on selected genes.** To determine gene set functional enrichment, over-representation testing was performed using the ConsensusPathDB service utilizing a hypergeometric test over a large collection of pathways and gene ontology (GO) terms. The centrality metrics, which each had 24 genes, produced FDR corrected significant pathways and GO term results (Table 2). The KEGG cell cycle pathway was frequently found significant, and cell cycle related GO terms were found, but in low ranking positions. Several metrics, including alpha centrality and PageRank, returned poor results, with no cell cycle related terms found significant. Chromatin remodeling and metabolic related pathways and GO terms were often found significant, emphasizing the close relationship between the cell cycle, metabolism, and chromatin remodeling (S2 Table).

Among cell cycle related GO terms, we found that different metrics contained different functional relations. In cell cycle chronological order, Ego 2-step and Betweenness both had ‘regulation of transcription involved in G1 phase of mitotic cell cycle’, while ranked influential genes showed ‘regulation of transcription involved in G1/S phase of mitotic cell cycle’ (GO:0000083). Betweenness showed significance with ‘regulation of transcription involved in G2/M-phase of mitotic cell cycle’ (GO:0000117).
